# Cutaneous *Mycobacterium chelonae* Infection During Ibrutinib Treatment in Chronic Lymphocytic Leukemia: A Case Report

**DOI:** 10.3390/microorganisms14061189

**Published:** 2026-05-25

**Authors:** Serena Bergamo, Giusto Trevisan, Giovanna Muffato, Diana Sacchi, Serena Bonin, Alessandro Gatti

**Affiliations:** 1 Dermatology Department, AULSS 2 Marca Trevigiana, Ospedale Ca’ Foncello, 31100 Treviso, Italy; serena.bergamo@aulss2.veneto.it (S.B.); alessandro.gatti@aulss2.veneto.it (A.G.); 2 Department of Medical Sciences, University of Trieste, 34151 Trieste, Italy; sbonin@units.it; 3 Microbiology Department, Ospedale Ca’ Foncello, 31100 Treviso, Italy; giovanna.muffato@aulss2.veneto.it; 4 Department of Pathology, Azienda ULSS2 Marca Trevigiana, 31100 Treviso, Italy; diana.sacchi@aulss2.veneto.it

**Keywords:** *Mycobacterium chelonae*, B-cell chronic lymphocytic leukemia, ibrutinib

## Abstract

*Mycobacterium chelonae* is a rapidly growing nontuberculous mycobacterium (NTM) that can infect both immunocompetent and immunocompromised hosts. Cutaneous and soft tissue infections are the most common manifestations and occur more frequently in individuals with underlying immune dysfunction. Patients with chronic lymphocytic leukemia (CLL), particularly those receiving targeted therapies such as ibrutinib, may be at increased risk of opportunistic infections. The diagnostic workup, microbiological findings, antimicrobial susceptibility testing, and therapeutic approach adopted for a cutaneous *M. chelonae* infection arising in a CLL patient four months after the introduction of ibrutinib were described. Clinical course and surgical management are also reported. A 60-year-old beekeeper with B-cell CLL developed a progressive cutaneous lesion on the left lower limb within four months of starting ibrutinib. Culture of a skin biopsy identified *M. chelonae*. Antimicrobial therapy was initiated based on in vitro susceptibility testing, resulting in partial clinical improvement. Complete resolution required surgical excision of the infected tissue followed by skin grafting. The patient’s underlying hematologic disease, ongoing immunosuppression, and recent exposure to ibrutinib likely contributed to susceptibility and persistence of infection. This case highlights the increasing recognition of nontuberculous mycobacterial infections in immunocompromised individuals and underscores the importance of early diagnosis and susceptibility- guided therapy. Clinical response may be incomplete, and combined medical and surgical approaches may be required in selected cases. NTM infections should be considered in patients receiving Bruton’s tyrosine kinase inhibitors who present with persistent, atypical, or non-healing cutaneous lesions. However, the association between ibrutinib therapy and susceptibility to infection remains uncertain, as multiple predisposing factors may coexist. Increased awareness of this possible association, together with careful clinical evaluation, may facilitate earlier diagnosis and improved management.

## 1. Introduction

*Mycobacterium chelonae* (Mc) is a non-motile, non-spore-forming, Gram-positive, acid-fast, nonchromogenic, rapidly growing mycobacterium (RGM), belonging to the group of nontuberculous mycobacteria (NTM). It is classified in Group IV of the Runyon classification and was first isolated in 1903 by Freidmann from lung tissues of sea turtles (*Chelona corticata*). The genus name derives from the Greek words mykēs (fungus), baktērion (small rod), and chelōnē (turtle). RGMs are divided into six groups: the *Mycobacterium fortuitum* group, the *M. chelonae*/*M. abscessus* complex, the *M. smegmatis* group, the *M. mucogenicum* group, *M. mageritense*/*M. wolinskyi*, and the pigmented RGM group [1]. Mc is part of the *M. chelonae*/*M. abscessus* complex, and can be distinguished from *M. abscessus* by differences in the intergenic sequence (ITS) region; notably, Mc is characteristically susceptible to tobramycin, in contrast to *M. abscessus* [2]. Mc is ubiquitous in soil, water, aquatic animals, and drinking water distribution systems, as it is resistant to chlorine-based disinfection [3,4]. This environmental resilience contributes to its persistence in both community and healthcare settings, where it has been associated with nosocomial outbreaks linked to contaminated medical devices, solutions and surgical instruments [5,6]. Reported cases occur worldwide, with an overall increasing recognition of NTM infections in recent years [3]. No age, sex, or ethnic predilection has been identified, and person-to-person transmission has not been documented [7].

Clinically, Mc frequently causes skin and soft tissue infections (cellulitis, abscesses) [8], particularly on the extremities, where lower temperatures may favor bacterial proliferation. Lesions may evolve into pustules, hemorrhagic crusts, or abscesses and can follow trauma or invasive procedures. Ocular involvement represents the second most common manifestation [9]. Additional presentations include catheter-related infections and post-surgical infections following implants, kidney transplantation [10,11], endoscopy, laparoscopy [12], sclerotherapy, tattooing [13], dermal fillers injection [14], and acupuncture [15]. Sporotrichoid lymphocutaneous disease may occur [4], and disseminated disease is typically restricted to immunosuppressed hosts. Sweet’s syndrome has also been described as an associated manifestation [16].

Diagnosis relies on molecular techniques such as PCR [17], and PCR-restriction analysis (PRA) targeting heat shock protein genes [18]. Culture and antimicrobial susceptibility testing remain essential to guide therapy, as Mc, although usually sensitive to macrolides and aminoglycosides, may exhibit variable and sometimes unpredictable resistance patterns [19]. Here, we report the case of cutaneous *M. chelonae* infection involving the left calf and ankle in a patient with chronic lymphocytic leukemia (CLL), arising shortly after the initiation of ibrutinib therapy.

## 2. Case Report

**Figure 1:**
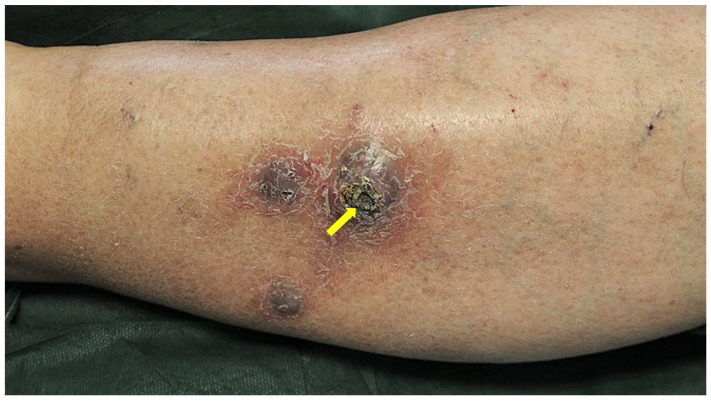
Skin lesion on the left calf before initiation of antibiotic therapy. The yellow arrow indicates the site of sampling.

A 60-year-old beekeeper presented with violaceous, firm, painful, and swollen nodules on the left leg and lateral aspect of the ankle (Figure 1). His medical history included stage B B-cell chronic lymphocytic leukemia (CLL), according to Binet classification, with secondary hypogammaglobulinemia, type 2 diabetes mellitus, chronic obstructive pulmonary disease with bronchiectasis and recurrent pulmonary infections, obesity, obstructive sleep apnea syndrome, hypertension, and atrial fibrillation associated with ibrutinib therapy. For CLL, he had previously received standard chemotherapy; rituximab and idelalisib were discontinued due to disease progression. Before initiation of ibrutinib, complete blood count showed: white blood cells 3.7 × 10^3^/μL (neutrophils 47%, lymphocytes 37%, monocytes 10%, eosinophils 6%), red blood cells 5.28 × 10^6^/µL, and platelets 120 × 10^3^/µL. The patient started ibrutinib therapy in August 2019, achieving good clinical control. In December 2019, ulcerated, painful erythematous–violaceous nodular lesions developed on the left calf and ankle (Figure 1). Biopsy specimens were obtained from the affected skin for histopathological and microbiological evaluation.

**Figure 2:**
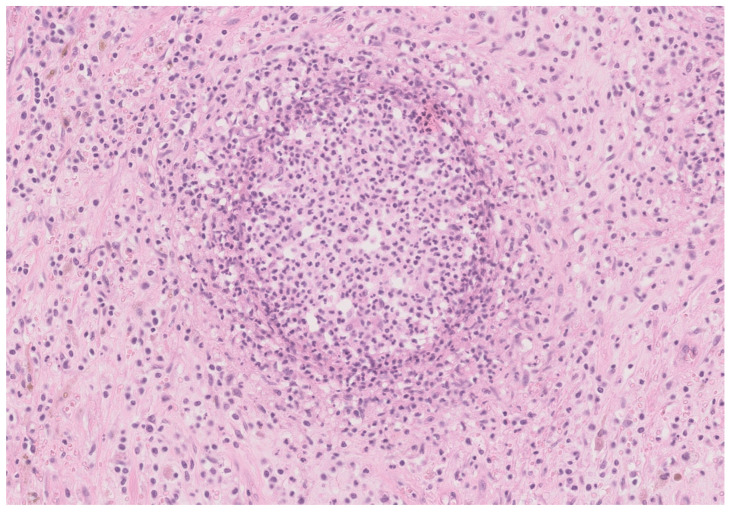
Non-necrotizing epithelioid granuloma with central neutrophilic infiltration and a peripheral rim of lymphocytes (200× magnification).

Histopathological analysis (Figure 2) revealed a mixed inflammatory infiltrate with peripheral epithelioid histiocytes, consistent with a suppurative granulomatous reaction, and numerous central neutrophils, a pattern commonly associated with atypical cutaneous mycobacterial infections in both immunocompromised and immunocompetent patients. Ziehl-Neelsen staining revealed the presence of acid-fast bacilli in the tissue.

Cultures for mycobacteria were performed using liquid medium (Mycobacterial Growth Indicator Tube, MGIT, Becton, Dickinson and Company, Franklin Lakes, NJ, USA), solid media (Löwenstein-Jensen, Becton, Dickinson and Company, Franklin Lakes, NJ, USA), and Middlebrook 7H11 agar (Becton, Dickinson and Company, Franklin Lakes, NJ, USA) incubated at 30 °C. Species identification was performed after 25 days of culture and achieved by MALDI-TOF mass spectrometry (Bruker Daltonik GmbH, Bremen, Germany), with subsequent confirmation by DNA probe-based assays (GenoType Mycobacterium CM and GenoType NTM-DR, Hain Lifescience GmbH, Nehren, Germany). Sequencing was not performed. No other cases of *M. chelonae* were identified in the laboratory during the same period, allowing us to reasonably exclude possible contamination. DNA strip testing for macrolide- and aminoglycoside-resistance genes was also performed. The initial biopsy culture was positive for *Mycobacterium chelonae*. The DNA-STRIP assay for genotypic resistance to macrolides showed no mutations in the *erm* and/or *rrl* genes, and the assay for aminoglycoside resistance showed no mutations in the *rrs* gene. An antibiogram of *M. chelonae* was also performed, demonstrating susceptibility to doxycycline (MIC = 0.25 µg/mL). The complete results are shown in Table 1.

Both standard bacterial and mycobacterial blood cultures, as well as sputum cultures and chest CT, showed no evidence of systemic dissemination. Based on antimicrobial susceptibility testing, a multidrug regimen was initiated under infectious disease supervision. The patient received intravenous imipenem for two weeks, followed by intravenous amikacin (1 g, three times per week- Monday, Wednesday, and Friday) for two months, linezolid (600 mg once daily) for two months, azithromycin (500 mg once daily), and doxycycline (100 mg twice daily) for four months. Doxycycline was included in the treatment regimen based on the antibiogram results and on available clinical evidence suggesting that this agent may contribute to the efficacy of combination therapies used to treat rapidly growing nontuberculous mycobacterial infections [20,21]. However, tetracyclines (including doxycycline) often appear ineffective against slow-growing nontuberculous mycobacteria [22], and further randomized controlled trials are needed to better define their role. Regular monitoring was performed, including twice-weekly assessment of serum creatinine, complete blood count, and alanine aminotransferase (ALT) levels, as well as weekly evaluation of vestibular and auditory function, ophthalmologic examination, and electrocardiography to monitor the QT interval. No significant adverse effects occurred. After four months of therapy, partial improvement of cutaneous lesions was observed, but complete resolution was not achieved. A second biopsy remained positive for *M. chelonae*, again without detectable resistance genes.

**Figure 3:**
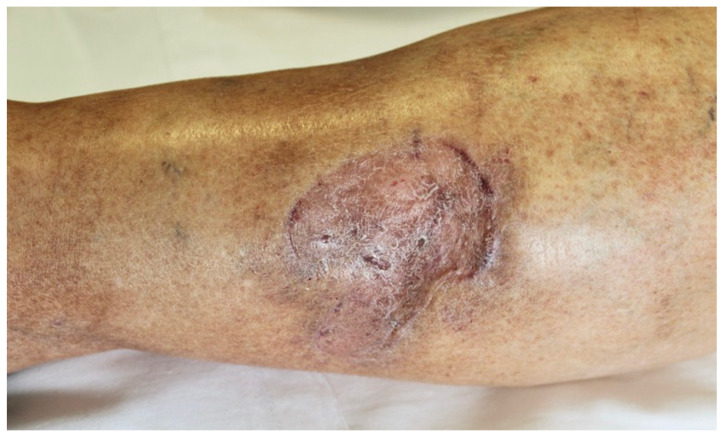
Left calf six months after surgical excision and skin grafting.

Given the persistence of lesions, a plastic surgery consultation was obtained. Surgical excision with skin grafting was performed, and postoperative cultures were negative. Antibiotic therapy was discontinued three months after surgery, with no evidence of recurrence (Figure 3).

At six-month follow-up after completion of antibiotic therapy (October 2020), microbiological culture performed on a biopsy specimen obtained from the lesion site was negative. Hematological evaluation showed a complete blood count with white blood cells of 22,140/μL (neutrophils 75.8%, lymphocytes 20.4%, monocytes 2.3%, eosinophils 1.2%, basophils 0.4%), red blood cells 4.80 × 10^6^/µL, Hemoglobin 14.9 g/dL and platelets 140 × 10^3^/µL. Serum creatinine was 1.10 mg/dL. The patient was continuing ibrutinib therapy (two tablets daily), with a good clinical response. On physical examination, no abnormal lung sounds were detected, but palpable lymph nodes in the axillary and inguinal area. The liver and spleen were palpable 2 cm below the costal margin. Cardiac examination revealed normal heart sounds with a regular rhythm. Serum protein electrophoresis showed total proteins of 6.2 g/dL, with the following distribution: albumin 63.2%, α1-globulin 4.3%, α2-globulin 11.1%, β1-globulin 7.2%, β2-globulin 4.2%, and γ-globulin 10.0%. Immunoglobulin levels were: IgG 681 mg/dL, IgA 26 mg/dL, and IgM 17.6 mg/dL. No monoclonal components were detected. The patient remains under follow-up for the hematologic condition, and no recurrence has been observed as of February 2026.

## 3. Discussion

*Mycobacterium chelonae* is a rapidly growing non tuberculous mycobacterium commonly found in the environment, particularly in water, aquatic animals, and soil. It can survive under harsh conditions and cause a variety of infections, most frequently involving the skin and soft tissues. The patient’s occupational exposure as a beekeeper may also be relevant. Beekeeping involves frequent outdoor activities and direct contact with environmental reservoirs such as soil, water, and vegetation, all of which are recognized sources of nontuberculous mycobacteria, including *M. chelonae*. In addition, minor skin trauma, insect stings, or repeated micro-injuries to exposed areas may facilitate inoculation of the organism. Although no clear history of significant trauma or contaminated water exposure was documented in this case, the possibility of occupational inoculation to the affected limb cannot be excluded. These considerations highlight the importance of carefully exploring environmental and occupational exposures in patients presenting with cutaneous NTM infections.

Infections due to *M. chelonae* may be asymptomatic, but most commonly present with cutaneous and soft tissue manifestations, as observed in the present case. They occur more frequently in immunosuppressed individuals, although cases in immunocompetent hosts have also been reported. Pulmonary involvement is uncommon and is mainly described in patients with cystic fibrosis [23]. In immunosuppressed patients, including those receiving corticosteroids, monoclonal antibodies, or post-transplant immunosuppressive therapy, as well as in individuals with malignancy or chronic kidney disease, *M. chelonae* may cause invasive or disseminated disease. Disseminated cutaneous infection is typically characterized by multiple papules and pustules, often involving proximal extremities, the face and the upper trunk [24]. Other reported manifestations described in the literature include myositis [25], spondylitis [26], arthritis, sepsis [27], intra-abdominal abscess, and mediastinal infection [2]. In the present case, ibrutinib was considered a key predisposing factor. However, the patient also had several additional risk factors for *M. chelonae* infection, including chronic lymphocytic leukemia (CLL), secondary hypogammaglobulinemia, type 2 diabetes mellitus, chronic lung disease with bronchiectasis, recurrent infections, obesity, and prior chemoimmunotherapy. Each of these conditions may independently increase susceptibility to nontuberculous mycobacterial infections. Cases of NTM infection in patients with CLL receiving ibrutinib have been reported. In particular, one case described infection with the RGM *M. chelonae*; unlike the present case, that patient developed thrombocytopenia following antibiotic treatment [28]. Another report described a patient with CLL treated with ibrutinib who developed infection with *Mycobacterium avium*, a slow-growing NTM [29]. The clinical presentation is heterogeneous, ranging from localized cutaneous involvement to disseminated disease, and may be associated with variable treatment responses. Compared with previously published cases, our patient showed a localized cutaneous infection but required prolonged antimicrobial therapy and surgical management, highlighting the potential for a persistent clinical course even in the absence of systemic dissemination. However, the limited number of reported cases underscores the need for further studies to better define the true incidence and optimal management of NTM infections in this setting.

Ibrutinib inhibits Bruton’s tyrosine kinase (BTK), a key component of B-cell receptor signaling [30], and has been shown to impair bacterial clearance by reducing γδ T cell activation and CD107a degranulation, potentially affecting bacterial clearance [31]. Similarly, *M. chelonae* infection has been reported following treatment with alemtuzumab, a humanized anti-CD52 monoclonal antibody targeting lymphocytes and monocytes [32,33]. Interestingly, ibrutinib has also been associated with a potential beneficial effect in *Mycobacterium tuberculosis* (Mtb) infection, as it may inhibit intracellular bacterial growth in macrophages through modulation of autophagy pathways, including reduced p62 expression and increased LC3b levels [34].

*Mycobacterium chelonae* exhibits variable antimicrobial susceptibility, and treatment should therefore be individualized based on in vitro susceptibility testing in accordance with current guideline recommendations [35,36]. Although this species is often reported to be susceptible to agents such as amikacin, macrolides, linezolid, and, less consistently, imipenem, doxycycline, and clofazimine [37,38], these generalizations should be interpreted with caution. Treatment failures and recurrences are not uncommon [39], often reflecting multidrug resistance [39] or the emergence of resistance during therapy. Macrolides, particularly clarithromycin and azithromycin, are generally considered a cornerstone of therapy when susceptibility is confirmed; however, resistance may develop and compromise clinical outcomes. This may occur through point mutations in the *23S rRNA* gene (e.g., at position 2058) [40]. Importantly, a distinction should be made between acquired resistance and inducible resistance mechanisms. Unlike *Mycobacterium abscessus*, in which inducible macrolide resistance mediated by *erm* genes is common, *M. chelonae* has traditionally been considered to lack functional *erm* genes, although recent evidence suggests that plasmid-mediated *erm* genes may confer inducible resistance in a minority of isolates [41,42]. These findings further emphasize the importance of susceptibility-guided therapy and careful microbiological monitoring during treatment. In refractory cases, surgical management, including debridement or excision with skin grafting, may be required, as in the present patient [43,44]. Additional therapeutic options for resistant infections include γ-interferon [45], omadacycline [8], and bacteriophage therapy [46]. Overall, treatment typically requires prolonged multidrug regimens administered over several weeks or months.

## 4. Conclusions

*Mycobacterium chelonae* is an emerging pathogen increasingly recognized as a cause of skin and soft tissue infections, particularly in immunocompromised hosts. This case highlights how underlying hematologic disease and treatment with ibrutinib may predispose patients to the development and progression of infection. Although antimicrobial therapy guided by susceptibility testing remains the cornerstone of management, clinical response may be incomplete, and combined medical and surgical approaches may be required in selected cases. Early recognition, prompt microbiological diagnosis, and careful therapeutic monitoring are essential to achieve clinical cure and prevent dissemination or recurrence. Growing evidence suggests that nontuberculous mycobacterial infections should be considered in patients receiving Bruton’s tyrosine kinase inhibitors who present persistent, atypical or non-healing cutaneous lesions. Increased awareness of this association, together with a multidisciplinary approach, may facilitate earlier diagnosis and more effective management of these challenging infections.

## Figures and Tables

**Table 1:** Antibiogram results for *Mycobacterium chelonae*.

Antibiotics	Interpretation	MIC (µg/mL)
Amikacin	Susceptible	8
Cefoxitin	Resistant	128
Ciprofloxacin	Susceptible	1
Clarithromycin	Susceptible	0.5
Doxycycline	Susceptible	0.25
Imipenem	Intermediate	16
Linezolid	Susceptible	8
Moxifloxacin	Susceptible	0.5
Tobramycin	Susceptible	<1

## Data Availability

The original contributions presented in this study are included in the article. Further inquiries can be directed to the corresponding author.

## Abbreviations

The following abbreviations are used in this manuscript:

BTK	Bruton’s tyrosine kinase
CLL	Chronic lymphocytic leukemia
Mc	Mycobacterium chelonae
Mtb	Mycobacterium tuberculosis
NTM	Nontuberculous mycobacteria
RGM	Rapidly growing mycobacterium
PCR	Polymerase Chain Reaction
